# Fine Mapping of *QUICK ROOTING 1* and *2*, Quantitative Trait Loci Increasing Root Length in Rice

**DOI:** 10.1534/g3.117.300147

**Published:** 2017-12-26

**Authors:** Yuka Kitomi, Emari Nakao, Sawako Kawai, Noriko Kanno, Tsuyu Ando, Shuichi Fukuoka, Kenji Irie, Yusaku Uga

**Affiliations:** * Institute of Crop Science, National Agriculture and Food Research Organization, Tsukuba, Ibaraki 305-8518, Japan; † Department of International Agricultural Development, Graduate School of Agriculture, Tokyo University of Agriculture, Setagaya-ku 156-8502, Japan

**Keywords:** CSSLs, *Oryza sativa*, root elongation, root system architecture, QTLs

## Abstract

The volume that the root system can occupy is associated with the efficiency of water and nutrient uptake from soil. Genetic improvement of root length, which is a limiting factor for root distribution, is necessary for increasing crop production. In this report, we describe identification of two quantitative trait loci (QTLs) for maximal root length, *QUICK ROOTING 1* (*QRO1*) on chromosome 2 and *QRO2* on chromosome 6, in cultivated rice (*Oryza sativa* L.). We measured the maximal root length in 26 lines carrying chromosome segments from the long-rooted upland rice cultivar Kinandang Patong in the genetic background of the short-rooted lowland cultivar IR64. Five lines had longer roots than IR64. By rough mapping of the target regions in BC_4_F_2_ populations, we detected putative QTLs for maximal root length on chromosomes 2, 6, and 8. To fine-map these QTLs, we used BC_4_F_3_ recombinant homozygous lines. *QRO1* was mapped between markers RM5651 and RM6107, which delimit a 1.7-Mb interval on chromosome 2, and *QRO2* was mapped between markers RM20495 and RM3430-1, which delimit an 884-kb interval on chromosome 6. Both QTLs may be promising gene resources for improving root system architecture in rice.

For terrestrial plants, the root is an essential organ capturing water and nutrients from the surrounding soil. Many wild plants have evolved root systems that are able to adapt to various environmental stresses (Canadell *et al.* 1996). For example, under drought conditions, drought-resistant plants tend to have deep root systems and can thus avoid drought stress by absorbing water from subsoil. In crops, root system adaptations to a range of environments, which differ from region to region, may be beneficial for improving productivity (de Dorlodot *et al.* 2007; Uga *et al.* 2015a). In maize (*Zea mays* L.), a deep root system would be favorable for rapid acquisition of water and nitrogen from subsoil, because water and the nitrogen dissolved in it move downward owing to gravity (Lynch 2013). Saengwilai *et al.* (2014) reported that maize accessions with few crown roots take up more nitrogen from low-nitrogen soils than accessions with many crown roots. In rice (*Oryza sativa* L.), high-yielding cultivars often have higher root biomass in the deep soil layers of paddy fields in comparison with low-yielding varieties (Kawata *et al.* 1978; Morita *et al.* 1986, 1988). However, breeding for root traits has progressed slowly because phenotypic selection is more laborious and time consuming for root traits than for aboveground traits. Therefore, marker-assisted selection for quantitative trait loci (QTLs) without phenotypic selection may be a promising breeding strategy for improving root system architecture (de Dorlodot *et al.* 2007; Yamamoto *et al.* 2014).

The vertical distribution of root systems in monocotyledonous crops such as maize, wheat, and rice is determined mainly by root growth angle and the length of seminal and crown roots (Abe and Morita 1994; Araki *et al.* 2002). In rice, several QTLs for root growth angle have been detected or fine-mapped (Uga *et al.* 2011, 2012, 2013b, 2015b; Kitomi *et al.* 2015; Lou *et al.* 2015). Among them, *DEEPER ROOTING 1* (*DRO1*) on chromosome 9 has been cloned (Uga *et al.* 2013a). The mapping population was derived from a cross between the shallow-rooted lowland cultivar IR64 and the deep-rooted upland cultivar Kinandang Patong (Uga *et al.* 2013a). Kinandang Patong has a functional allele of *DRO1*, whereas IR64 has a nonfunctional allele. Field experiments using a near-isogenic line (Dro1-NIL), which carried the Kinandang Patong allele of *DRO1* in the IR64 genetic background, demonstrated that alteration of root system architecture from shallow to deep enhances drought avoidance and increases yields under drought stress (Uga *et al.* 2013a).

Several genes that affect root length have been identified in rice through mutant analyses. Most mutants have short roots as a result of repressed cell division, cell elongation, or both, and have abnormalities of genes involved in cell wall construction [*OsDGL1* (Qin *et al.* 2013), *OsMOGS* (Wang *et al.* 2014), *ROOT GROWTH INHIBITING*/*OsGLU3* (Inukai *et al.* 2012; Zhang *et al.* 2012)] and auxin signaling [*OsARF12* (Qi *et al.* 2012)]. Because all the reported mutant alleles reduce root length, they cannot be used for improving the efficiency of water and nutrient acquisition or early seedling establishment. On the other hand, favorable alleles of >100 QTLs for root length, as summarized by Courtois *et al.* (2009), might improve root system architecture in rice. Among these, a few QTLs have been narrowed down to small chromosome regions, although no QTLs for root length have been isolated in rice so far. Under hydroponic conditions, *qRL6.1*, a QTLs for root length detected at the seedling stage, was delimited to a 337-kb interval on chromosome 6 (Obara *et al.* 2010), and *qRL7*, a QTLs for root length detected at the heading stage, was mapped to a 657-kb interval on chromosome 7 (Wang *et al.* 2013). Thus, only a few genes can be considered promising at this stage for increasing root length and root growth angle in rice.

*DRO1* affects root growth angle but has no marked effect on root length or number, shoot length, or tiller number (Uga *et al.* 2013a). Therefore, QTLs for root length are needed to further improve root system architecture in rice; the efficiency of water and nutrient acquisition could be improved by combining such QTLs with *DRO1*. In this study, we measured maximal root length of rice chromosome segment substitution lines (CSSLs) grown in hydroponic culture to detect chromosomal regions increasing maximal root length. Next, we conducted rough mapping of five such regions. Finally, we fine-mapped two candidate QTLs.

## Materials and Methods

### Plant materials

For identification of genomic regions associated with maximal root length in rice, we used 26 CSSLs derived from a cross between IR64 and Kinandang Patong (IK-CSSLs in the IR64 genetic background; Uga *et al.* 2015b). IR64 is a modern lowland cultivar (*indica*; IRGC 66970) developed by the International Rice Research Institute in the Philippines, which is widely grown in South and Southeast Asia. Kinandang Patong is a traditional upland cultivar (*tropical japonica*; IRGC 23364) that originated in the Philippines. In the IK-CSSLs, each chromosome was covered by one to four lines that carried overlapping chromosomal segments, although small regions on chromosomes 2, 5, 7, 8, and 10 were not covered (Uga *et al.* 2015b).

For the QTLs analyses in five candidate regions associated with maximal root length in IK-CSSLs, we used five BC_4_F_2_ populations derived from self-pollinated BC_4_F_1_ plants with heterozygous target chromosomal regions, which had been selected during IK-CSSL development (Uga *et al.* 2015b). The BC_4_F_2_ for SL1006, SL1011, SL1015, SL1016, and SL1019 were #09-6642-34 (*n* = 144), #09-6647-13 (*n* = 79), #15-6456-7 (*n* = 119), #09-6651-10 (*n* = 74), and #09-S19-1 (*n* = 80), respectively.

For fine mapping of QTLs for maximal root length on chromosomes 2, 6, and 8, we developed homozygous BC_4_F_3_ lines in which recombination had occurred within the region containing the target QTLs. The number of BC_4_F_3_ line pairs for mapping of QTLs on chromosomes 2, 6, and 8 were 6, 16, and 7, respectively. Their BC_4_F_4_ progenies were used to determine the genotype of each QTLs in these lines.

### Measurement of maximal root length in hydroponic culture

We grew IR64, Kinandang Patong, and IK-CSSLs in hydroponic culture. Seeds were washed in sterilized water three times and sterilized in 1% PPM (plant preservative mixture; Plant Cell Technology, Inc., Washington, DC) at 15° in the dark for 1 d. Sterilized seeds were imbibed at 30° in the dark for 1.5 d. Uniform seedlings were selected and transferred onto a plastic net supported by a polystyrene plate floating on a hydroponic solution in a plastic tub (L × W × H = 54.4 cm × 34.4 cm × 26.0 cm). Each polystyrene floater included 12 rows with 10 or 12 seeds per row; each tub contained up to four floaters. Seeds were spaced 1.0 cm within and between rows. Tubs were wrapped in foil and filled with 43 liters of 1/4-strength Kimura B solution [91.25 µM (NH_4_)_2_SO_4_, 22.75 µM K_2_SO_4_, 136.75 µM MgSO_4_, 45.75 µM KNO_3_, 91.25 µM Ca(NO_3_)_2_, 45.5 µM KH_2_PO_4_, 4.45 µM FeC_6_H_5_O_7_·H_2_O] with 5 mM MES (C_6_H_13_NO_4_S·H_2_O) in a greenhouse. The final pH of hydroponic solution was adjusted to 5.5. We renewed the nutrient solution every 2 d, at which time we rerandomized tubs in the greenhouse and floaters in the tubs. The average air temperature was 30° during the day and 26° at night; the average relative humidity was 50%; plants were illuminated during the daytime from 6:00 to 18:00 by 400 W metal-halide lamps (M400DL/BUDP; Iwasaki Electric Co., Ltd., Tokyo, Japan). At approximately the two-leaf-stage of IR64 used as a control (7–9 d after sowing), the plants were removed from the plastic net, and the lengths of roots and shoots were measured with a ruler. The difference in the day of measurement was due to differences in plant growth rate between different cultivation periods. A randomized block design with two replications (12 seedlings per replicate) was used for evaluation of maximal root length in the IK-CSSLs.

### DNA marker analysis

The genotypes of BC_4_F_2_ and BC_4_F_3_ plants were determined by using simple sequence repeat (SSR) markers selected from the International Rice Genome Sequencing Project (2005) data and sequence-tagged site (STS) markers selected from Uga *et al.* (2008). After measurement of root and shoot lengths, a leaf was collected from each plant, and total leaf genomic DNA was extracted in 300 μl extraction buffer containing 1 M KCl, 100 mM Tris-HCl (pH 8.0), and 10 mM ethylenediamine tetraacetic acid (EDTA; pH 8.0). DNA was precipitated with 125 μl isopropanol, washed with 200 μl 70% ethanol, and dissolved in 100 μl 1/10 TE buffer containing 1.0 mM Tris-HCl (pH 8.0) and 0.1 mM EDTA (pH 8.0). Polymerase chain reaction (PCR) amplification for SSR analysis was performed in 5-µl reaction mixtures containing 1.0 µl DNA, 2.5 µl of KAPA2G Fast Ready Mix with dye (Kapa Biosystems, Boston, MA), 0.125 µl of a mixture of forward and reverse primers (20 µM each), and 1.375 μl H_2_O. The PCR program consisted of initial denaturation for 1 min at 95°; 35 cycles of 10 sec at 95°, 10 sec at 55°, and 1 sec at 72°; and a final extension for 30 sec at 72°. PCR products were separated by electrophoresis in 3% agarose gels (Type I-A, low EEO; Sigma-Aldrich, St. Louis, MO) at 200 V for 80 min.

### Statistical and QTLs analyses

To identify chromosomal regions responsible for maximal root length in IK-CSSLs, we used Dunnett’s test in JMP v. 11.2 software (SAS Institute, Cary, NC). All lines were compared with IR64 as the reference; 24 plants per line were assessed (12 plants per row × 2 replicates).

Construction of a linkage map and QTLs analysis for the five BC_4_F_2_ populations were performed in R/qtl software (http://www.rqtl.org/; Broman *et al.* 2003). The markers were ordered based on their physical map positions in the latest version of the Rice Annotation Project (RAP) database (IRGSP-1.0; http://rapdb.dna.affrc.go.jp/). Genetic distances were estimated using the software’s Kosambi map function (Kosambi 1944). Putative QTLs were detected using the composite interval mapping (CIM) function. The CIM threshold was based on the results of 1000 permutations at the 5% significance level (Churchill and Doerge 1994).

For fine mapping, we genotyped the same SSR and STS markers used in the QTLs analyses, and used additional SSR markers to saturate the regions around the putative QTLs detected in QTLs analyses. We compared the phenotypic values between two BC_4_F_4_ homozygous recombinant lines having homozygous alleles of IR64 or Kinandang Patong. The genotype of each line was estimated from the results of a student’s *t*-test at the 0.1% significance level in JMP v. 11.2. The sample size per BC_4_F_4_ line was 40 plants (10 plants per row × 4 replicates).

### Data availability

Supplemental Material, File S1 contains genotypes and phenotypes of the IK-CSSLs and BC_4_F_2_ populations used in this study.

## Results

### Maximal root length in IK-CSSLs grown under hydroponic conditions

**Figure 1:**
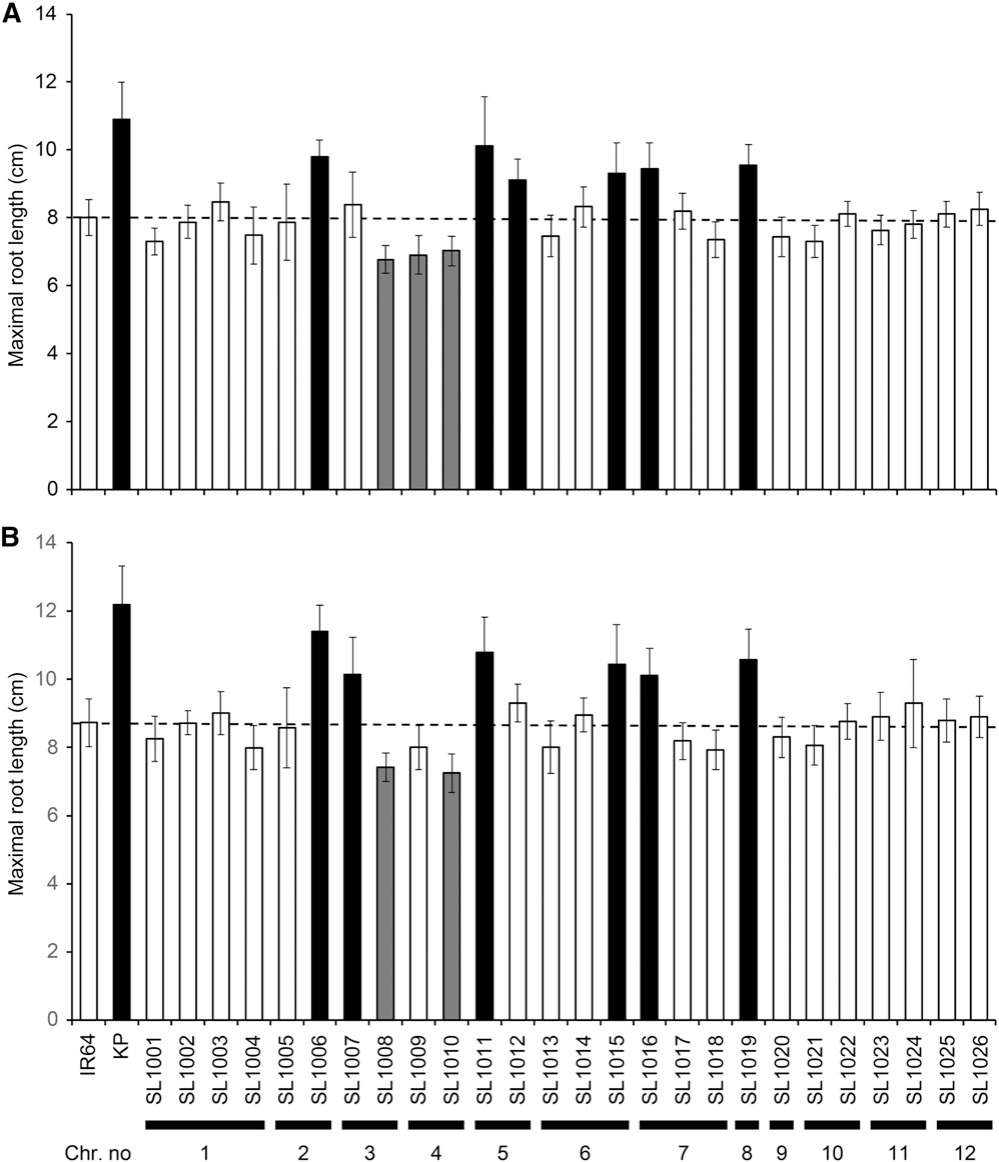
Maximal root lengths of 26 IK-CSSLs, IR64, and Kinandang Patong (KP) grown in hydroponic conditions. Values are given as mean ± SD (*n* = 24). Black and gray bars indicate lines with longer and shorter roots, respectively, than those of IR64 (*P* < 0.001, Dunnett’s test). Dashed lines show the mean values of IR64. Substituted chromosome in each line is indicated at the bottom. (A) First trial (data at 9 d after germination). (B) Second trial (data at 8 d after germination).

Kinandang Patong had longer roots than IR64 in two trials (Figure 1). We also confirmed that this phenotypic difference persisted from the early seedling stage until 6 wk after sowing (Figure S1). These results indicate that the floating hydroponic system allows evaluation of the natural variation in rice root length. In the first trial, six CSSLs (SL1006, SL1011, SL1012, SL1015, SL1016, and SL1019) had significantly longer roots than IR64, whereas three CSSLs (SL1008–SL1010) had significantly shorter roots (Figure 1A). In the second trial, six CSSLs (SL1006, SL1007, SL1011, SL1015, SL1016, and SL1019) had significantly longer roots than IR64, whereas two CSSLs (SL1008 and SL1010) had significantly shorter roots (Figure 1B). These trials indicated that five CSSLs (SL1006, SL1011, SL1015, SL1016, and SL1019) carried robust QTL(s) whose Kinandang Patong alleles increase maximal root length. We also measured shoot length to clarify its association with maximal root length in IK-CSSLs. None of the CSSLs with longer roots than IR64 had significantly longer shoots, although SL1007, SL1012, and SL1015 had shorter shoots than IR64 (Figure S2).

### Detection of QTLs for maximal root length

**Figure 2:**
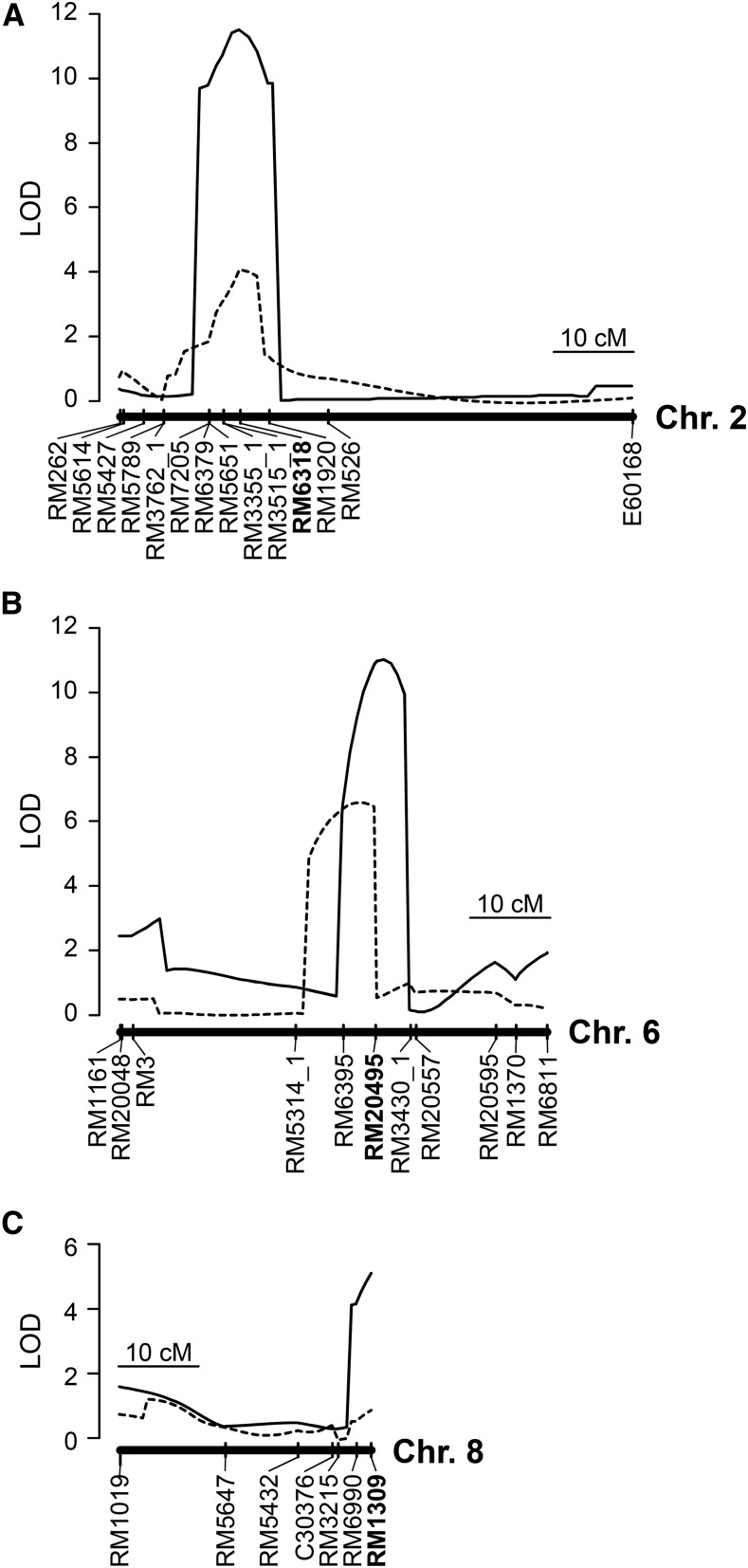
LOD score curves of QTLs for root and shoot lengths in the three BC_4_F_2_ mapping populations. Peaks indicate the putative QTLs positions. Solid lines indicate maximal root length; dotted lines indicate shoot length. Vertical ticks indicate the genetic positions (cM) of DNA markers. DNA markers closest to LOD peaks are shown in bold. (A) Population #09-6642-34 segregating for the long arm of chromosome 2; (B) population #15-6456-7 segregating for the long arm of chromosome 6; (C) population #09-S19-1 segregating for chromosome 8.

To validate the presence of QTLs associated with maximal root length in the five CSSLs selected, we conducted QTLs analyses in five BC_4_F_2_ mapping populations segregating for target chromosomal regions. We detected a QTLs for maximal root length on each of chromosomes 2, 6, and 8 (Figure 2), but not on chromosomes 5 or 7 (data not shown), at the logarithm of the odds (LOD) threshold based on the results of 1000 permutations at the 5% significance level.

**Figure 3:**
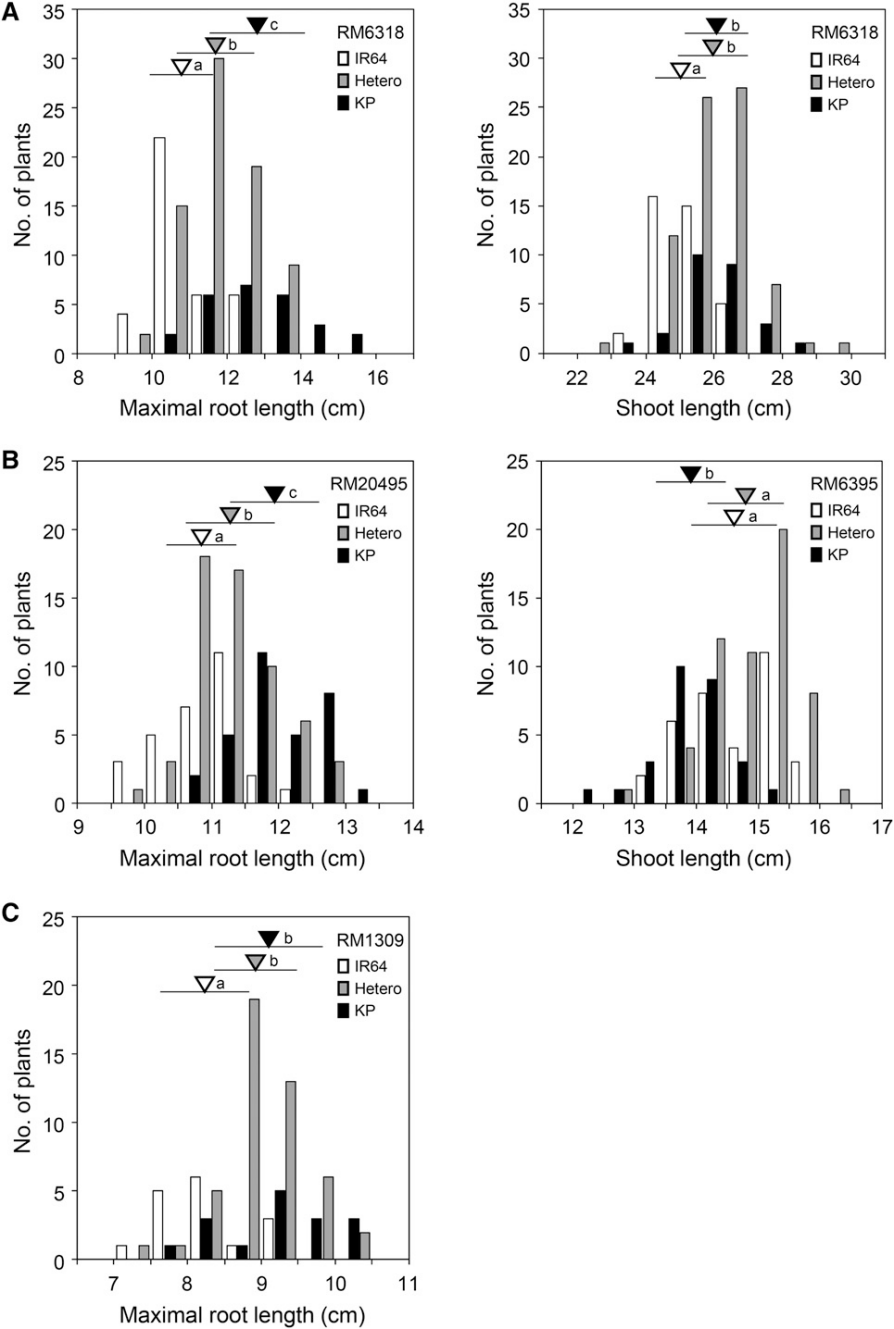
Frequency distributions of root and shoot lengths in the three BC_4_F_2_ mapping populations, showing three genotype classes of the DNA markers closest to the detected QTLs. For each allele, an inverted triangle indicates the mean, and a horizontal bar indicates SD. The same shading is used for triangles and corresponding bars. The means labeled with different letters differ significantly (*P* < 0.05, Tukey’s multiple comparison test). (A) Population #09-6642-34 segregating for the long arm of chromosome 2; (B) population #15-6456-7 segregating for the long arm of chromosome 6; (C) population #09-S19-1 segregating for chromosome 8.

One significant LOD peak (11.51) for maximal root length was detected at RM6318 on chromosome 2 (Figure 2A). This QTLs explained 30.7% of the total phenotypic variance (Table 1). The additive effect (*AE*) of the homozygous Kinandang Patong allele at this QTLs on maximal root length was 2.06 cm (*i.e.*, 2× the *AE* for a single copy of the allele). The mean maximal root lengths of lines homozygous for the Kinandang Patong allele at RM6318 were significantly larger than those of lines homozygous for the IR64 allele (Figure 3A). We also found a QTLs for shoot length at RM6318, although the shoot length of SL1006 was not significantly different from that of IR64 (Figure 2A and Table 1). Lines homozygous for the Kinandang Patong allele at RM6318 had significantly longer shoots than lines homozygous for the IR64 allele (Figure 3A).

**Table 1:** Putative QTLs for root and shoot length detected in BC_4_F_2_ populations

Population	Trait	Chr.	Nearest Marker	Mb*^a^*	cM*^b^*	LOD	Sh*^c^*	*AE**^d^*	*DE**^e^*	*R*^2^*^f^*
09-6642-34	Root length	2	RM6318	24.43	0.0	11.51	2.64	1.03	−0.13	30.7
	Shoot length	2	RM6318	24.43	0.0	4.07	2.95	0.50	0.44	16.5
15-6456-7	Root length	6	RM20495	26.55	1.4	11.04	2.66	0.56	−0.17	30.7
	Shoot length	6	RM6395	26.00	2.6	6.56	2.73	−0.36	0.60	26.7
09-S19-1	Root length	8	RM1309	19.18	0.0	4.85	2.75	0.46	0.24	21.4

a Physical map position of each marker based on the latest version of the RAP database (http://rapdb.dna.affrc.go.jp/).

b Genetic distance from the QTLs LOD peak to the nearest marker.

c Threshold of LOD value based on 1000 permutation tests at the 5% level.

d Additive effect of the allele from Kinandang Patong compared with that from IR64.

e Dominance effect of the allele from Kinandang Patong compared with that from IR64.

f Percentage of the phenotypic variance explained by each QTLs.

One significant LOD peak (11.04) for maximal root length was detected near RM20495 on chromosome 6 (Figure 2B). This QTLs also explained 30.7% of the total phenotypic variance (Table 1). The *AE* of the homozygous Kinandang Patong allele at this QTLs on maximal root length was 1.12 cm. The mean maximal root lengths of lines homozygous for the Kinandang Patong allele at RM20495 were significantly larger than those of lines homozygous for the IR64 allele (Figure 3B). We also found a QTLs for shoot length near RM6395 (Figure 2B and Table 1). Lines homozygous for the Kinandang Patong allele at RM6395 had significantly shorter shoots than lines homozygous for the IR64 allele (Figure 3B), suggesting that this QTLs may be associated with the shorter shoots of SL1015 than of IR64.

One significant QTLs for maximal root length was detected at RM1309 on chromosome 8, but no significant QTLs for shoot length was found in the same region (Figure 2C). This QTLs explained 21.4% of the total phenotypic variance (Table 1). The *AE* of the homozygous Kinandang Patong allele at this QTLs on maximal root length was 0.92 cm. The mean maximal root lengths of lines homozygous for the Kinandang Patong allele at RM1309 were significantly greater than those of lines homozygous for the IR64 allele (Figure 3C).

### Fine mapping of maximal root length QTLs

To map the three QTLs for maximal root length detected on chromosomes 2, 6, and 8, we used three BC_4_F_3_ populations in which recombination occurred within the target region of each chromosome.

**Figure 4:**
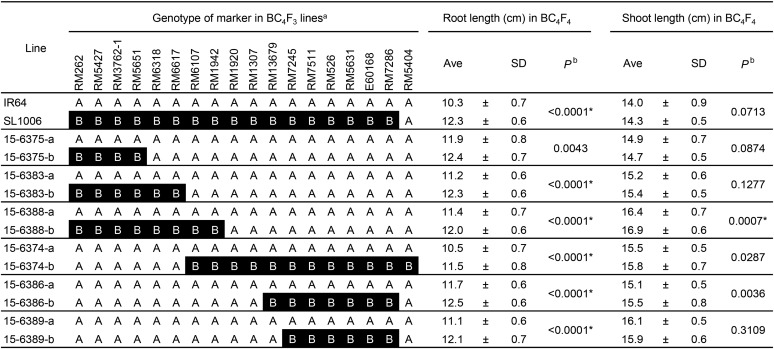
Genotypes of 18 DNA markers on chromosome 2 in BC_4_F_3_ lines and phenotypes of their progeny (BC_4_F_4_). ^a^A (white background), IR64 homozygous; B (black background), Kinandang Patong homozygous. ^b^Probability of no significant difference between lines in a pair (student’s *t*-test). *Significance at the 0.1% level.

**Figure 5:**
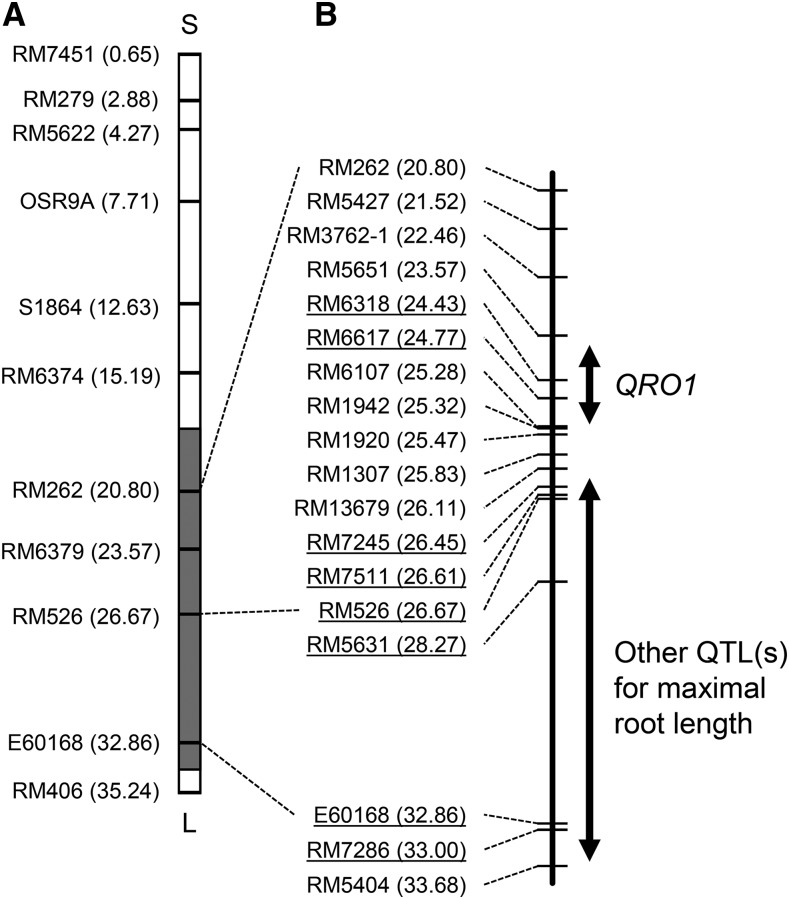
Location of *QRO1* on chromosome 2. (A) Graphical genotype of SL1006. White and gray boxes represent regions homozygous for the IR64 and Kinandang Patong alleles, respectively. S indicates short arm; L indicates long arm. (B) Physical map position of *QRO1*. Double-headed arrows indicate candidate regions of *QRO1* and other putative QTLs for maximal root length. DNA markers are shown on the left side of each map; numbers in parentheses indicate physical map positions of the markers in the latest version of the RAP database. Underlines indicate no recombination with the predicted genotype of *QRO1*.

For the QTLs on chromosome 2, significant differences in maximal root length, ranging from 0.6 to 1.1 cm, between lines were found in all line pairs except #15-6375 (Figure 4), whereas the roots of SL1006 were 2.0 cm longer than those of IR64. These results suggest that this QTLs alone does not fully explain the total phenotypic variance in SL1006, and that two or more QTLs in this region are involved. On the basis of this hypothesis, we compared the genotypes of BC_4_F_3_ lines and the phenotypes of their BC_4_F_4_ progeny and identified two chromosomal regions related to maximal root length: one between RM5651 (23.57 Mb) and RM6107 (25.28 Mb), and the other between RM13679 (26.11 Mb) and RM5404 (33.68 Mb) (Figure 5). We designated the maximal root length QTLs in the former region *QUICK ROOTING 1* (*QRO1*). On the other hand, we could not narrow down the latter region sufficiently as a single locus. A significant difference in shoot length was found only in one pair of lines, #15-6388, demonstrating that the shoot length QTLs on chromosome 2 was located between RM6617 (24.77 Mb) and RM6107 (25.28 Mb), *i.e.*, within the candidate region of *QRO1*.

**Figure 6:**
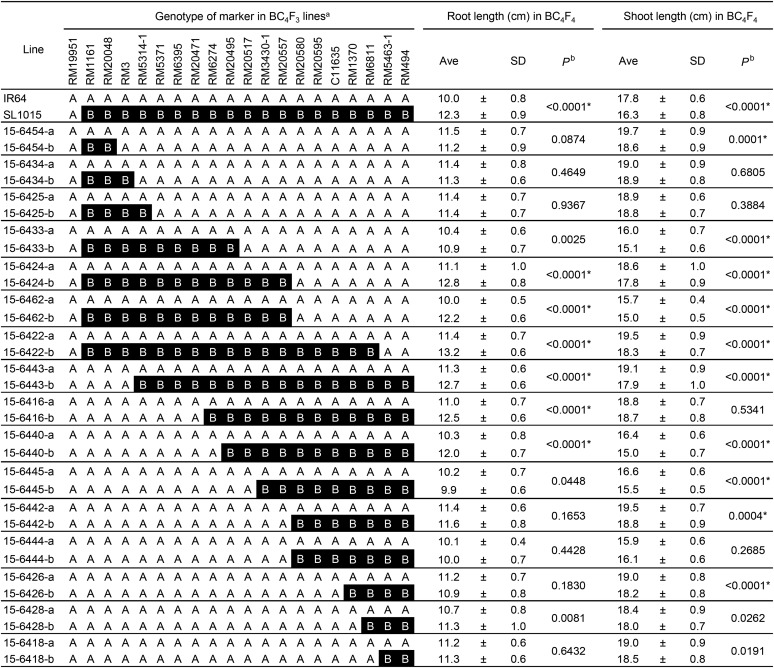
Genotypes of 20 DNA markers on chromosome 6 in BC_4_F_3_ lines and phenotypes of their progeny (BC_4_F_4_). ^a^A (white background), IR64 homozygous; B (black background), Kinandang Patong homozygous. ^b^Probability of no significant difference between lines in a pair (student’s *t*-test). *Significance at the 0.1% level.

**Figure 7:**
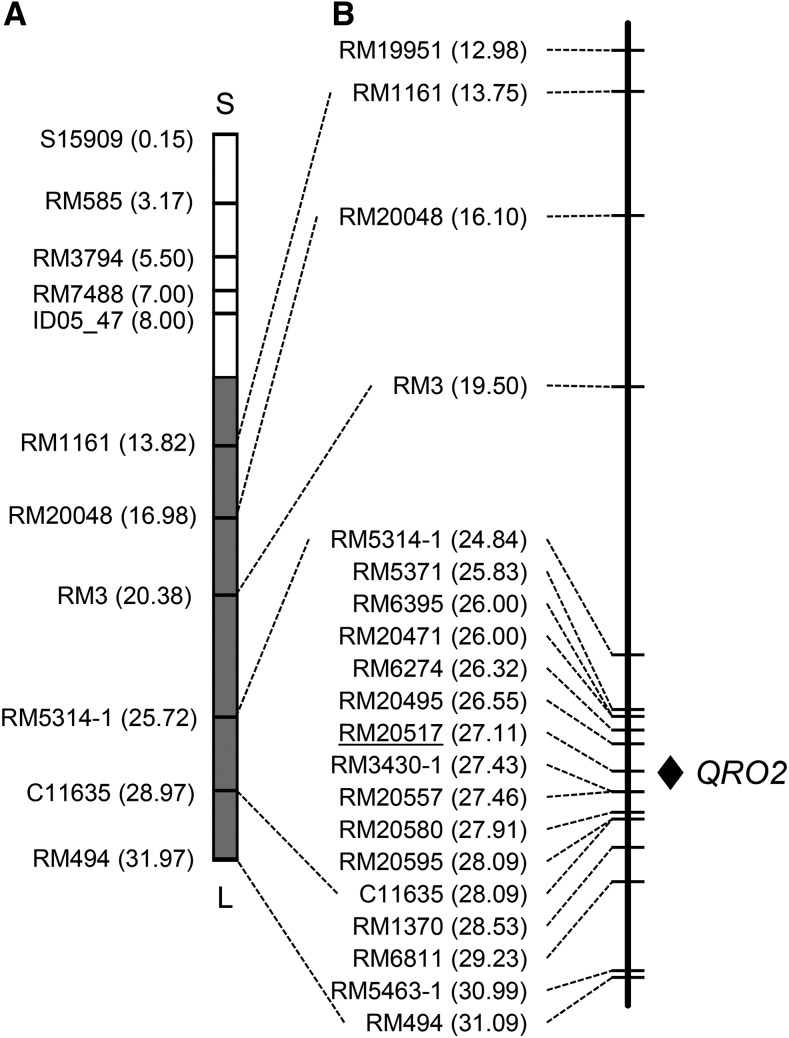
Location of *QRO2* on chromosome 6. (A) Graphical genotype of SL1015. White and gray boxes represent regions homozygous for the IR64 and Kinandang Patong alleles, respectively. S indicates short arm; L indicates long arm. (B) Physical map position of *QRO2*. A short double-headed arrow indicates the candidate region of *QRO2*. DNA markers are shown on the left side of each map; numbers in parentheses indicate physical map positions of the markers in the latest version of the RAP database. Underline indicates no recombination with the predicted genotype of *QRO2*.

For the QTLs on chromosome 6, significant differences in maximal root length were found in the line pairs #15-6424, #15-6462, #15-6422, #15-6443, #15-6416, and #15-6440 (Figure 6), indicating that the QTLs is located between RM20495 (26.55 Mb) and RM3430-1 (27.43 Mb) (Figure 7). We designated this QTLs as *QUICK ROOTING 2* (*QRO2*). We could not identify the specific region of the QTLs for shoot length by comparing the genotypes of BC_4_F_3_ lines and BC_4_F_4_ phenotypes.

For the QTLs on chromosome 8, significant differences in maximal root length were detected in all line pairs except #15-6518 and #15-6519 (Figure S3). Comparison between the genotype and phenotype data suggested that SL1019 might have two QTLs for maximal root length in different regions: between C30376 and RM6215 and between RM5351-1 and RM23229. We were unable to narrow down the candidate region of the target QTLs in this mapping population, as both intervals were very wide.

## Discussion

We analyzed the phenotypes of 26 CSSLs and identified five CSSLs that had longer roots at the seedling stage because of the presence of chromosome segments from Kinandang Patong. These results indicate that several regions, including those on chromosomes 2, 5, 6, 7, and 8, are associated with the difference in maximal root length between IR64 and Kinandang Patong. Furthermore, the possible presence of multiple QTLs on chromosomes 2 and 8 suggests that maximal root length is controlled by multiple genes on the same chromosome. For such a trait, the development and use of mapping populations with a simple genetic background such as CSSLs may become a powerful tool to reliably detect QTLs.

We narrowed down the candidate regions of two QTLs for maximal root length on chromosomes 2 (1.7 Mb) and 6 (884 kb) by fine mapping, and designated these QTLs as *QRO1* and *QRO2*, respectively (Figure 5 and Figure 7). Next, we surveyed the presence of genes and QTLs related to root length reported by previous studies in the *QRO1* and *QRO2* candidate regions in three online databases: Overview of Functionally Characterized Genes in Rice Online (OGRO, http://qtaro.abr.affrc.go.jp/ogro/table; Yamamoto *et al.* 2012), QTLs Annotation Rice Online (Q-TARO, http://qtaro.abr.affrc.go.jp/qtab/​table, Yonemaru *et al.* 2010), and RAP-DB (http://rapdb.dna.affrc.go.jp; Sakai *et al.* 2013). No genes or QTLs related to root length were found in OGRO or Q-TARO in the 1.7-Mb *QRO1* candidate region. In RAP-DB, among ∼200 genes predicted in this region, we found cell division-related genes such as those encoding cyclins and transcription factors, including *MYB*s. In the 884-kb *QRO2* candidate region, we found no genes or QTLs in OGRO or Q-TARO. In RAP-DB, ∼120 genes, including those encoding an auxin efflux carrier and several transcription factors, are predicted in this region. These genes might be candidates for *QRO1* and *QRO2*. To identify the causative genes of *QRO1* and *QRO2*, we are now performing high-resolution mapping. Comparison of the regions of two QTLs on chromosome 2 detected in this study and meta-QTL for root length reported by Courtois *et al.* (2009) showed that both QTLs regions included meta-QTL, suggesting that two regions may have a robust effect on root length in different genetic backgrounds if they are identical to these meta-QTL. On the other hand, Courtois *et al.* (2009) reported no hot spot of QTLs for root length on chromosome 6. Obara *et al.* (2010) fine-mapped such a QTLs (*qRL6.1*) on the long arm of chromosome 6, but its location is distinct from that of *QRO2*. These comparisons indicate that *QRO2* is likely a novel QTLs for root length.

Seminal root elongation immediately after germination is a valuable trait for any crop, because it accelerates access to water and nutrients, resulting in the early establishment of vigorous seedlings (Rogers and Benfey 2015). *QRO1* and *QRO2* certainly promote quick seminal root elongation for a given length of time after germination. In *Arabidopsis*, mutants with a larger root meristem tend to have longer roots, and root meristem size is regulated by the balance between the plant hormones auxin and cytokinin (Dello Ioio *et al.* 2007, 2008). However, these studies measured root growth rate but not the maximal root length. Maximal root length may depend on cell division activity in the root apical meristem, but no report has identified genes contributing to the maximal root growth in any plant species. Therefore, whether maximal root length correlates with root meristem size or root growth rate remains unknown. To assess the durability of root growth promotion by *QRO1* and *QRO2*, we will investigate their effects on maximal root length using QRO1-NIL and QRO2-NIL in greenhouse and field trials.

In the field, lower soil layers contain more water owing to gravity. The grain yield of Dro1-NIL, which carries a functional allele of *DRO1* derived from Kinandang Patong in the genetic background of IR64, was higher than that of IR64 under drought conditions, because Dro1-NIL had deeper rooting and could obtain more water from lower soil layers than IR64 (Uga *et al.* 2013a). Dro1-NIL also had a higher grain yield than IR64 in paddy fields, because deeper rooting enhances nitrogen uptake from lower soil layers (Arai-Sanoh *et al.* 2014). On the other hand, the maximal root length of Dro1-NIL was identical to that of IR64, because *DRO1* controls mainly root growth angle (Uga *et al.* 2013a). If *QRO1* and/or *QRO2* increase maximal root length, rice plants possessing their functional allele(s) could obtain water and nutrients under a wide range of field conditions. Crossing Dro1-NIL with QRO1-NIL and/or QRO2-NIL may enable development of rice plants with a greater capacity to obtain water and nutrients from deeper soil layers than that of Dro1-NIL. To develop QRO2-NIL, we have to separate *QRO2* from the QTLs for short shoots on chromosome 6 to ensure vigorous early growth, if these QTLs are derived from respective genes (Figure 2B). We expect that the use of these root-related genes may allow us to improve the rice root system, resulting in high yields.

## Supplementary Material

Supplemental material is available online at www.g3journal.org/lookup/suppl/doi:10.1534/g3.117.300147/-/DC1.
