# Supplementary material for: Fine Mapping of QUICK ROOTING 1 and 2, Quantitative Trait Loci Increasing Root Length in Rice
Source: G3 (Bethesda). 2017 Dec 26;8(2):727–35. doi: 10.1534/g3.117.300147 (PMC5919730; doi:10.1534/g3.117.300147)
Supplement: Supplementary file 1 [file 727FigureS1.pdf]

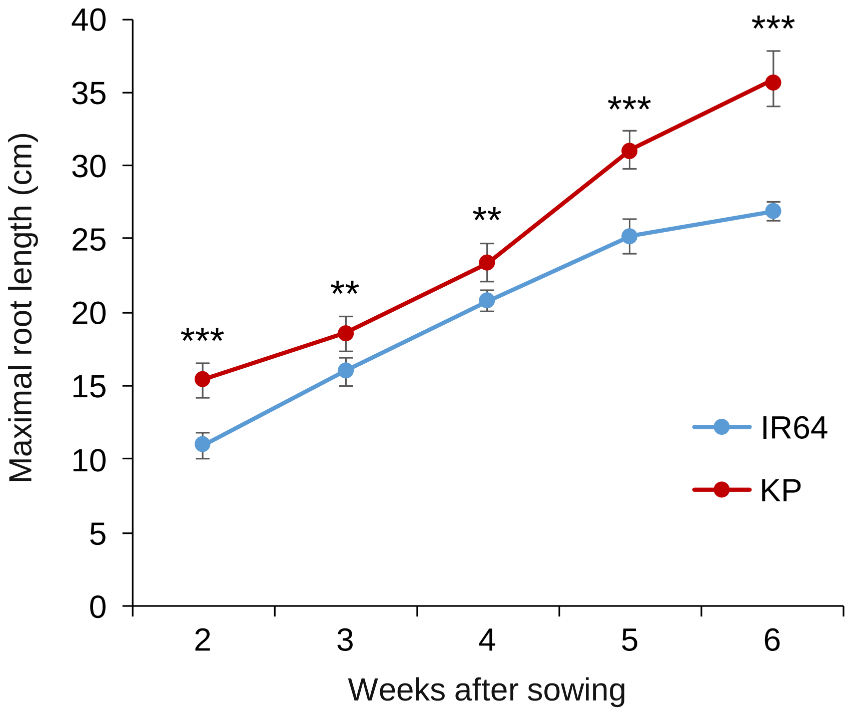

Figure S1. Time course of root growth in IR64 and Kinandang Patong (KP) in hydroponic media. Plot shows mean ± s.d. (n = 5). **Significant at the 1% level; ***significant at the 0.1% level (Student's *t*-test). Maximal root length was measured as described in Uga et al. (2010).

Reference:

Uga, Y., K. Okuno, and M. Yano, 2010 Fine mapping of *Sta1*, a quantitative trait locus determining stele transversal area, on rice chromosome 9. Mol. Breed. 26: 533–538.
