# Supplementary material for: Fine Mapping of QUICK ROOTING 1 and 2, Quantitative Trait Loci Increasing Root Length in Rice
Source: G3 (Bethesda). 2017 Dec 26;8(2):727–35. doi: 10.1534/g3.117.300147 (PMC5919730; doi:10.1534/g3.117.300147)
Supplement: Supplementary file 2 [file 727FigureS2.pdf]

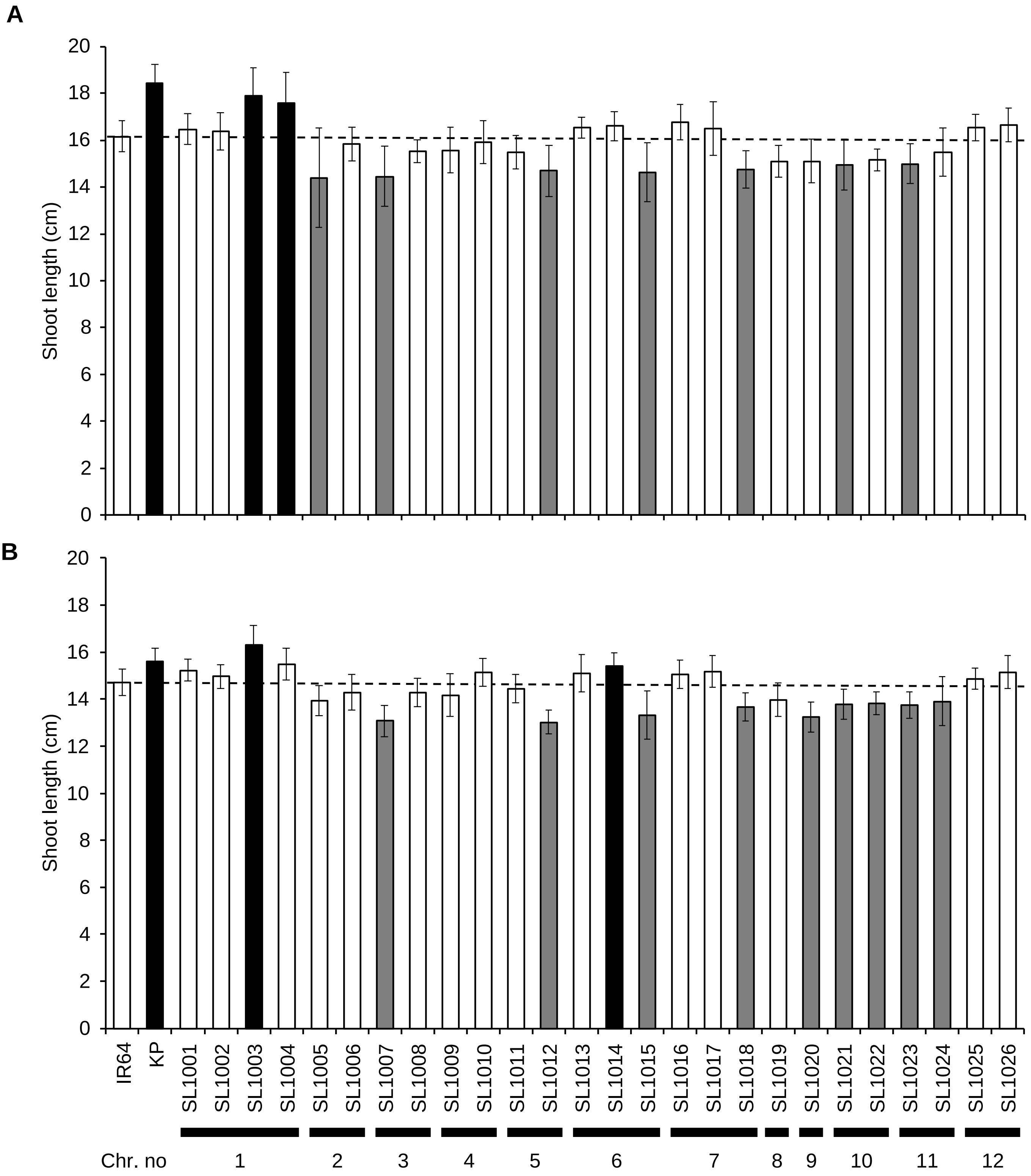


**Figure S2.** Shoot lengths of 26 IK-CSSLs, IR64, and Kinandang Patong (KP) grown in hydroponic conditions. Values are means ± SD ($n$ = 24). Black and gray bars indicate lines with longer and shorter roots than those of IR64, respectively ($p$ < 0.001, Dunnett's test). Dashed lines show the mean values of IR64. Substituted chromosome in each line is indicated at the bottom. (**A**) 1st trial (data at 9 days after germination). (**B**) 2nd trial (data at 8 days after germination).
