# Supplementary material for: Fine Mapping of QUICK ROOTING 1 and 2, Quantitative Trait Loci Increasing Root Length in Rice
Source: G3 (Bethesda). 2017 Dec 26;8(2):727–35. doi: 10.1534/g3.117.300147 (PMC5919730; doi:10.1534/g3.117.300147)
Supplement: Supplementary file 3 [file 727FigureS3.pdf]

Line	Genotype of marker in BC_4F_3 lines[a]																				Root length in BC_4F_4			Shoot length in BC_4F_4		
	C30376	RM3215	RM3481	RM3662	RM6990	RM22934	RM6215	RM1309-1	RM3689	RM3459	RM5351-1	RM5637	RM7356	RM23201	RM23204	RM23211	RM23214	RM23217	RM23229	RM6635	Ave	SD	P^b	Ave	SD	P^b
IR64	A	A	A	A	A	A	A	A	A	A	A	A	A	A	A	A	A	A	A	A	9.6	± 0.5	<0.0001*	13.8	± 0.8	0.004
SL1019	B	B	B	B	B	B	B	B	B	B	B	B	B	B	B	B	B	B	A	A	10.7	± 0.8		13.2	± 0.7	
15-6504-a	A	A	A	A	A	A	A	A	A	A	A	A	A	A	A	A	A	A	A	A	10.8	± 0.6	<0.0001*	14.2	± 0.6	0.1907
15-6504-b	B	B	B	B	B	B	A	A	A	A	A	A	A	A	A	A	A	A	A	A	11.7	± 0.6		14.0	± 0.6	
15-6515-a	A	A	A	A	A	A	A	A	A	A	A	A	A	A	A	A	A	A	A	A	12.0	± 0.7	<0.0001*	14.5	± 0.7	<0.0001*
15-6515-b	B	B	B	B	B	B	B	B	B	A	A	A	A	A	A	A	A	A	A	A	12.6	± 0.5		13.7	± 0.7	
15-6508-a	A	A	A	A	A	A	A	A	A	A	A	A	A	A	A	A	A	A	A	A	11.6	± 0.7	<0.0001*	14.0	± 0.5	0.3239
15-6508-b	B	B	B	B	B	B	B	B	B	B	B	A	A	A	A	A	A	A	A	A	12.5	± 0.6		13.9	± 0.6	
15-6518-a	A	A	A	A	A	A	A	A	A	A	A	A	A	A	A	A	A	A	A	A	11.0	± 0.5	0.8568	15.4	± 0.6	0.1432
15-6518-b	A	A	A	A	A	A	B	B	B	B	B	B	B	B	B	B	B	B	A	A	11.0	± 0.5		15.1	± 0.8	
15-6512-a	A	A	A	A	A	A	A	A	A	A	A	A	A	A	A	A	A	A	A	A	11.2	± 0.6	<0.0001*	15.6	± 1.0	0.1512
15-6512-b	A	A	A	A	A	A	A	B	B	B	B	B	B	B	B	B	B	B	A	A	11.9	± 0.7		15.3	± 0.7	
15-6521-a	A	A	A	A	A	A	A	A	A	A	A	A	A	A	A	A	A	A	A	A	10.7	± 0.5	<0.0001*	15.1	± 0.8	0.3746
15-6521-b	A	A	A	A	A	A	A	A	A	B	B	B	B	B	B	B	B	B	A	A	11.4	± 0.6		15.3	± 0.7	
15-6522-a	A	A	A	A	A	A	A	A	A	A	A	A	A	A	A	A	A	A	A	A	11.0	± 0.6	<0.0001*	15.4	± 0.7	0.4239
15-6522-b	A	A	A	A	A	A	A	A	A	A	A	B	B	B	B	B	B	B	A	A	11.6	± 0.7		15.3	± 0.6	
15-6519-a	A	A	A	A	A	A	A	A	A	A	A	A	A	A	A	A	A	A	A	A	11.9	± 0.5	0.0379	15.4	± 0.8	0.4932
15-6519-b	A	A	A	A	A	A	A	A	A	A	A	B	B	B	B	B	B	B	A	A	11.6	± 0.6		15.6	± 0.7	

Figure S3. Genotypes of 20 DNA markers on chromosome 8 in BC_4F_3 lines and phenotypes of their progeny (BC_4F_4). [a] A (white background), IR64 homozygous; B (black background), Kinandang Patong homozygous. [b]Probability of no significant difference between lines in a pair by Student's *t* test. * Significance at the 0.1% level.
